# Consumer ethnocentrism and political identity in value formation within public health communication for healthcare products: evidence from China

**DOI:** 10.3389/fpubh.2025.1693501

**Published:** 2026-01-14

**Authors:** Donger Zhang, Ruixia Ji, Sang-Do Park

**Affiliations:** 1 Department of International Commerce and Business, Konkuk University, Seoul, Republic of Korea; 2 Department of International Trade, Konkuk University, Seoul, Republic of Korea

**Keywords:** consumer ethnocentrism, credence goods, healthcare consumption, integrated TAM–VAM, political identity, public health communication, trust-based adoption model

## Abstract

**Background:**

Healthcare products are typical credence goods, for which it is difficult to completely verify quality and safety even after use. Therefore, consumers’ judgments depend greatly on trust and information credibility rather than cost. This study presents a trust-based model integrating the Technology Acceptance Model and the Value-based Adoption Model to explain the effects of message cues and sociocultural factors on consumers’ value formation and purchase intention within the institutional and social context shaped by China’s digital transformation, population aging, and the Healthy China 2030 policy framework.

**Methods:**

An online survey was conducted on 875 Chinese consumers through the Wenjuanxing platform. The research model was verified using structural equation modeling, and moderation–mediation analysis and multi-group analysis were performed to examine the moderating effects of consumer ethnocentrism and political identity (membership in the Chinese Communist Party, CCP).

**Results:**

The analysis showed that advertising perception, brand image, and personalization had significant positive effects on perceived quality, while price and information cues did not show direct effects. Perceived quality strongly improved perceived value, and perceived value appeared as the most decisive factor predicting purchase intention. Consumer ethnocentrism significantly moderated the relationship between quality and value, showing that culturally grounded trust strengthens value formation. In addition, the multi-group analysis results showed that the CCP member group had stronger relationships among perceived value, purchase intention, and the quality–value mediation path than the non-member group.

**Conclusion:**

This study extended TAM–VAM to the context of credence goods, highlighting that consumer decision-making in healthcare markets is driven less by functional utility and more by trust-based value formation mechanisms. It also empirically confirmed that consumer ethnocentrism and political identity are sociocultural moderating factors that strengthen the cognitive and emotional value transfer. From a practical and policy-oriented perspective, the findings suggest that healthcare communication and public health strategies should emphasize credibility-enhancing messages, transparent information disclosure, and culturally resonant personalization to foster preventive health consumption and reinforce public trust.

## 1 Introduction

Health-related consumer goods are typical credence goods, because consumers cannot easily verify their safety or efficacy either before or after use (1). As a result, purchase decisions for health products depend on message cues and trust—what kind of information is provided, who provides it, and how well it fits an individual’s health needs are decisive factors (2). In the field of public health, how such messages are designed and delivered is highly important, because effective communication can increase preventive uptake, improve adherence, and reduce exposure to misinformation (2, 3). Within this background, China provides a particularly prominent context. Rapid population aging, the growing burden of chronic diseases, and strong policy attention to prevention [e.g., Healthy China 2030 (4)] have expanded demand for digital health, functional foods, and other health products (4–6). At the same time, social media and live-commerce ecosystems have amplified both trustworthy information and low-quality noise (7). Therefore, understanding which message factors stably shape trust and value is not merely a marketing issue but a priority for public health.

Existing studies related to this topic have mainly explained the acceptance of health technologies and products through the Technology Acceptance Model (TAM) [e.g., (8–12)] and the value-based perspective [e.g., (13, 14)]. Most of these studies consistently demonstrated that perceived usefulness/quality and perceived value form behavioral intention. However, research gaps remain across three spectrums. First, previous studies often examined factors such as price, advertising, brand, or information quality individually and did not trace the complete chain of message cues → perceived quality → perceived value → purchase intention within the context of credence goods. Second, although personalization has been increasing across digital health, its role as a perceived quality signal distinct from general product attributes has not been clearly defined. Third, sociocultural factors are also important. Consumer ethnocentrism (preference for domestic products) and political identity may shape how quality cues are interpreted, but empirical evidence for these moderating variables in high-involvement health product purchases remains limited (15).

This study aims to address these gaps by integrating the TAM and the Value-based Adoption Model (VAM) and by including sociocultural moderating variables. We theorize that five message factors—price, advertising perception, brand image, information perception, and personalization—affect perceived quality (PQ), which in turn increases perceived value (PV) and subsequently influences purchase intention (PI). In addition, we assume that consumer ethnocentrism (CE) strengthens the relationship between PQ and PV, and we explore political identity (membership in the Chinese Communist Party, CCP) as a segmentation dimension through multi-group analysis (MGA). Empirically, the model is tested using PLS-SEM based on a large-scale online sample, with predictive validity verified in parallel.

The contributions of this study can be presented in three aspects. First, on the theoretical level, our research links message design to value formation in the context of credence goods, showing that transparent information, trustworthy branding, and personalization—rather than price—are the key drivers of PQ and PV. It also extends the role of CE from a simple preference to a moderating variable of cognitive evaluation and empirically demonstrates segmentation by political identity in the value formation of health products. Second, at the methodological level, this study applies PLS-SEM together with MGA and out-of-sample predictive evaluation, verifying both explanatory and predictive power. Finally, at the practical and public health level, the findings can be translated into communication strategies that improve preventive uptake and informed choice—for example, prioritizing the clarity and sufficiency of health information, signaling credibility through brand and expert endorsement, and emphasizing personalization functions tailored to individual conditions. Communication strategies can also be adjusted by audience segments differentiated by consumer ethnocentrism or political identity. In other words, by reconstructing message design as a form of public health intervention in the credence goods market, this study provides implications for health promotion, risk communication, and consumer protection efforts in China and similar national or market contexts.

## 2 Literature review

### 2.1 Healthcare products and consumer behavior theory

China’s demand for healthcare products has experienced structural expansion due to rapid population aging, the spread of chronic diseases, and the rising awareness of health management. At the macro level, health-related consumption and economic growth show a mutually reinforcing relationship. That is, regions that integrate healthcare investment closely with economic development exhibit higher levels of health expenditure (16, 17). This trend implies that it is important to understand not only consumers’ purchasing behavior but also the spillover effects that such behavior exerts on the overall public health system.

Studies on Chinese consumer behavior have mainly used the TAM and its extended form, the Unified Theory of Acceptance and Use of Technology (UTAUT), to explain the adoption of healthcare technologies and products. Empirical research, especially when focusing on middle-aged and older adults, reports that perceived usefulness (PU) and perceived ease of use (PEU) strongly predict the acceptance and continued use of wearable devices and online health services (18–20). Moreover, extended models that simultaneously consider user characteristics and device functions show higher explanatory power. However, healthcare consumption requires multidimensional evaluations that go beyond simple technology acceptance—covering efficacy, safety, time, cost, and trust.

To complement these limitations, the VAM has been introduced. VAM regards PV as the key determinant of behavioral intention. Related studies in China report that PV mediates or directly influences satisfaction, trust, emotional responses, and subsequently the intention to continue using or purchasing (14, 19). For example, research on private dental clinics found that service quality and brand image influence loyalty through the mediation of PV and satisfaction (21, 22). In addition, the reliability and clarity of health information strengthen purchase and continued-use intentions, while information overload and fatigue instead induce avoidance (23, 24).

In this way, TAM explains cognitive evaluations centered on technological utility, whereas VAM explains emotional and trust-based evaluations centered on value formation. Since healthcare products are new technology-oriented health goods that combine technological innovation with health management utility, it is reasonable to integrate the two models. Consumers perceive emerging healthcare products such as functional foods, nutritional supplements, and digital health devices not as ordinary goods but as new technological adoptions. In this context, the PU and PEU in TAM can be transferred and interpreted as health management efficiency and preventive benefits, respectively. These can explain actual healthcare adoption behaviors better than the attitudinal and normative factors mainly described by Theory of Reasoned Action (TRA) or Theory of Planned Behavior (TPB). Furthermore, TAM has a structural flexibility that can expansively integrate trust-related factors such as information credibility, brand reliability, and personalization, making it more suitable for explaining the psychological mechanisms of public health communication and preventive health consumption.

Therefore, we combine the value formation path of VAM with the technology acceptance path of TAM, exploring the continuous mechanism among PQ, PV, and PI. By internalizing the concepts of risk perception and trust dependence presented by the Health Belief Model (HBM) and the Extended Parallel Process Model (EPPM), this study reinterprets health-related consumption as a process of trust-based value adoption, rather than as simple technology acceptance (25, 26). Consequently, the integration of TAM and VAM is expected to provide a multi-layered framework that connects “health information trust – quality evaluation – value formation – behavioral intention” from a public health perspective.

### 2.2 Theoretical discussion on key variables

#### 2.2.1 Price perception

Price perception (PP) refers to the subjective evaluation in which consumers interpret a product’s price as a cue for quality, fairness, and value (27). In healthcare-related credence goods, a high price can serve as a proxy indicator of quality and safety, whereas a low price can lead to skepticism about authenticity or efficacy (28, 29). In the Chinese context, although the capacity for economic affordability is important, consumers have been reported to prioritize trust, credibility, and safety over cost (30, 31).

At this point, the direction of PP is influenced by perceived price fairness (the level deemed appropriate relative to cost and efficacy) and its position relative to the reference price (higher or lower compared to the category average). Depending on the activation level of the price–quality schema (high price = high quality), the same price signal can be interpreted in opposite ways (28, 29, 32, 33). In addition, the influence of PP can be amplified or weakened through interactions with brand trust, information credibility, and the trustworthiness and informativeness of advertising (for example, even a high price can be interpreted as a quality cue when the information is transparent and the brand is trusted).

Furthermore, there may be heterogeneity in the marginal effects depending on income level, sensitivity to health risk, and product involvement, or even nonlinear (inverted-U shaped) responses at extremely low or excessively high prices (30, 31). Therefore, price can substantially contribute to the formation of PQ, either directly or through interactions with other cues.

#### 2.2.2 Advertising perception

Advertising perception (AP) refers to consumers’ evaluation of the informativeness, credibility, and persuasiveness of advertising messages, which captures their judgment of trust and reliability regarding promotional messages and appeals (34). In the healthcare context, advertising functions not merely as exposure but as a factor shaping attitudes, and consumers respond more positively when advertisements are clear, accurate, and based on scientific evidence (35). Conversely, exaggerated or misleading claims weaken trust, increase skepticism, and, with repeated exposure, can cause information fatigue (23). This means that advertising perception operates as a variable that can exert multidimensional effects on perceived product quality.

Since COVID-19, China has undergone a comprehensive digital transformation, and various consumer behaviors are now conducted in digital environments. Healthcare products are no exception. Because the digital environment combines live-commerce with intensive promotional exposure, advertising perception functions as a crucial filter distinguishing trustworthy health information from commercial noise (36).

Furthermore, the informativeness and persuasiveness of advertising serve not only to promote consumption but also to strengthen the public trust of health-related messages (34). Trust-based communication enables consumers to interpret risk information more rationally, which enhances the stability of PQ and confidence in value perception. Therefore, in the field of healthcare products, advertising perception operates not merely as a marketing variable but as a psychological factor mediating the trust structure of health communication (23, 35).

#### 2.2.3 Brand image

Brand image (BI) refers to the set of associations, beliefs, and symbolic meanings that consumers connect with a specific brand, playing a central role in overall product evaluation and trust formation (37). Especially in credence good domains such as healthcare, brand image acts as a major heuristic cue that reduces uncertainty for consumers who find it difficult to directly verify product quality (38). Strong brands that convey reliability, expertise, and responsibility reduce perceived risk and induce positive quality evaluations (39).

In healthcare consumption, brand associations—such as perceived safety, clinical endorsement, or alignment with national standards—function as factors that strengthen trust and increase PI (22). Recently, in the Chinese market, the meaning of brand trust has been further reinforced through its combination with cultural factors. The spread of patriotic consumption leads domestic brands to be perceived not merely as economic choices but as symbols of national trust and authenticity, thereby amplifying the impact of brand image on quality evaluation and value judgment (40).

Consequently, BI enhances PQ, which in turn increases PV, ultimately influencing consumers’ PI through this sequential path. This process suggests that brand trust is not merely a marketing asset but a core factor in the mechanism of value formation for healthcare products.

#### 2.2.4 Information perception

Information perception (IP) refers to the cognitive evaluation of how accurate, sufficient, and useful product-related information is in reducing uncertainty during consumers’ decision-making processes (41). Unlike AP, which focuses on the formal credibility and persuasiveness of messages, information perception emphasizes the validity and clarity of the informational content itself. In other words, while AP concerns “who speaks and in what manner,” IP concerns “what is presented and how well it is substantiated.” When product information is accurate, transparent, and easy to process, consumers report higher levels of trust and confidence (24). Conversely, information overload, low reliability, or ambiguous information can undermine PQ and PV.

Research on public health communication highlights that clarity, sufficiency, and evidence-based messaging are key means of offsetting the impact of misinformation in such contexts (16, 42). Therefore, IP is a major cognitive factor that determines the PQ of healthcare products, and it is theoretically connected to how the accuracy of “perceived threat” and “efficacy information” affects behavioral intention, as presented in the HBM and the EPPM (25, 26, 43). In other words, IP functions as a cognitive assurance mechanism through which consumers rationalize their health-related decisions by verifying the reliability and scientific basis of information.

#### 2.2.5 Personal customization

Personal customization (PC) refers to the extent to which consumers perceive that marketing messages, recommendations, or services are adjusted to fit their own preferences, health needs, or situational contexts (44). This allows consumers to perceive greater relevance and control over information, thereby strengthening message acceptance and engagement. In the era of digital health, personalization acts not merely as a service adjustment but as a quality cue signaling the responsiveness and innovation of a platform (45). Such personalization makes consumers evaluate the product’s perceived fit and usefulness more highly when they feel that the provided information aligns with their own health goals. Empirical studies indicate that personalized health communication enhances PQ, reduces perceived risk, and increases satisfaction, thereby reinforcing PV (14).

In particular, China’s digital health platforms provide personalized health information and customized solutions through big data- and AI-based recommendation systems (46), and such data-driven personalization functions as a key factor in consumer trust and engagement (47). Therefore, personalization functions not merely as a marketing technique but as a health communication strategy for behavioral adherence that changes individuals’ health perceptions and behaviors.

#### 2.2.6 Perceived quality, perceived value, and purchase intention

PQ refers to consumers’ overall judgment of a product’s excellence and reliability and is closely associated with perceptions of trust and safety in evaluating healthcare products (29). Such quality perception reflects a psychological assurance in which consumers feel that risk is minimized and efficacy is guaranteed, rather than a mere functional assessment.

High PQ increases PV, which comprehensively represents the balance between benefits and sacrifices as perceived by consumers. Numerous studies have consistently identified PV as a mediating variable between PQ and purchase-related outcomes, showing the process by which consumers translate quality perception into trust-based value judgment (19, 22).

Finally, PI serves as the immediate behavioral outcome, primarily driven by PV, which is formed through consumers’ evaluations of PQ. Rich empirical evidence supports the sequential path PQ → PV → PI, emphasizing that consumer decision-making in credence-good markets such as healthcare products is not a simple comparison of cost and benefit but a trust-based value evaluation process rather than a purely functional assessment.

#### 2.2.7 Consumer ethnocentrism

CE is defined as a belief system in which consumers perceive purchasing domestic products as morally appropriate and economically beneficial, while viewing foreign alternatives as less desirable or disloyal (48). CE goes beyond simple economic nationalism; it reflects an emotional attachment to the in-group that drives trust and value formation in consumption (49). Numerous studies have confirmed that CE influences attitudes, PI, and brand evaluations, showing that the higher the level of CE, the stronger the preference for domestic products and the lower the openness to imported goods (48, 50). In the Chinese context, CE has been reported as a key factor shaping consumer decision-making across various markets such as food, apparel, and technology, influencing emotional responses, trust, and perceptions of authenticity (27).

In credence goods such as healthcare products, CE is particularly important. Because consumers find it difficult to directly verify product efficacy, they use the product’s national origin and identity as strong cues for quality (40). Therefore, CE functions as a cultural trust heuristic that strengthens the link between PQ and PV. The more consumers identify with their national institutions, the more symbolic and moral value they assign to domestic healthcare products. Such patriotic trust partially intersects with political identity within the context of public health policies such as China’s Healthy China 2030 initiative. Consequently, CE provides an important sociocultural foundation for trust formation and value judgment in health communication.

#### 2.2.8 Political identity (CCP membership)

Political identity can serve as a meaningful exploratory segmentation variable in consumer decision-making. In the Chinese context, membership in the CCP reflects alignment with state-led narratives, patriotism, and institutional trust in public health policies (51). Previous studies have reported that political orientation can shape perceptions of economic policy, institutional trust, and responsiveness to official communication (52).

Although political identity has been relatively underexplored in the field of healthcare consumption, it may influence how individuals interpret health-related message cues—particularly in their judgments of brand credibility and information reliability (53). Political identity is not merely an ideological distinction but functions as a sociopolitical lens through which consumers place trust in national institutions and public communication.

Party members are more likely to internalize government-endorsed public health messages as acts of civic duty and pursuit of collective good, thereby reinforcing both normative conformity and epistemic trust. As a result, political identity acts as a higher-order social influence factor that mediates institutional trust in the process through which perceptions of healthcare product quality are transferred into value and behavioral intention.

### 2.3 Research model and hypotheses

This study proposes a model that TAM, VAM, and trust–risk perception within the dimension of public health communication to explain consumers’ PI toward healthcare products. This is because consumers make judgments not only based on technological utility but also on trust and assurance under uncertainty and information asymmetry, and their decision-making can be explained as a combination of cognitive evaluation and emotional–social trust judgment.

**Figure 1:**
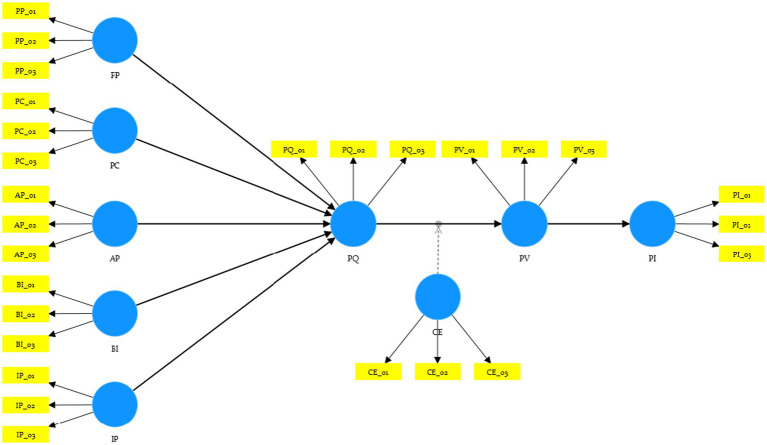
Research model and hypotheses.

Accordingly, we constructed a sequential path in which message cues—PP, AP, BI, IP, and PC—affect PQ, PQ influences PV, and PV mediates the effect on PI. CE is set as a cultural trust moderator that strengthens the relationship between PQ and PV, while political identity (CCP membership) is compared through MGA as an indicator of institutional trust. This integrated model simultaneously captures both the functional–cognitive dimension and the social–identity dimension of consumer behavior, explaining the process through which perceived quality and trust-based value formation lead to purchase intention for healthcare products. Figure 1 presents the conceptual model and hypothesized relationships, and Table 1 summarizes the main hypotheses.

**Table 1:** Summary of hypotheses.

No.	Hypothesis	Structural path	Expected direction
H1	Price perception positively affects perceived quality.	PP → PQ	+
H2	Advertising perception positively affects perceived quality.	AP → PQ	+
H3	Brand image positively affects perceived quality.	BI → PQ	+
H4	Information perception positively affects perceived quality.	IP → PQ	+
H5	Personal customization positively affects perceived quality.	PC → PQ	+
H6	Perceived quality positively affects perceived value.	PQ → PV	+
H7	Perceived value positively affects purchase intention.	PV → PI	+
H8	Consumer ethnocentrism strengthens the positive relationship between perceived quality and perceived value.	CE × PQ → PV	+(Moderation)
RQ1	Do structural relationships differ between CCP members and non-members?	MGA	Group difference

## 3 Methodology

### 3.1 Data collection

To empirically verify the proposed model, a survey was conducted in March 2025 through the online survey platform Wenjuanxing.^1^ Participants were recruited using Wenjuanxing’s panel-based invitation system, through which survey links were electronically distributed to eligible adult consumers on a voluntary basis. Prior to the main survey, to ensure the clarity and content validity of the questionnaire items, two experts in the fields of consumer studies and business/marketing reviewed the appropriateness of the items, and a pretest with 50 respondents was conducted to check the comprehensibility of the items and the consistency of responses. After incorporating feedback, the main survey was administered.

In total, 1,000 survey invitations were distributed, of which 875 valid responses were retained, yielding a response rate of 87.5%, after excluding insincere responses (careless response patterns, repeated selections of the same option) and duplicate submissions. The final sample size exceeds commonly recommended thresholds for partial least squares structural equation modeling (PLS-SEM) and ensures sufficient statistical power for estimating the proposed research model.

The survey was conducted entirely on a voluntary and anonymous basis. All respondents were informed in advance about the purpose of the study and provided informed consent prior to participation, and no personally identifiable or sensitive information was collected at any stage of the data collection process.

### 3.2 Measurement

All latent constructs were modeled as reflective and were revised and supplemented based on measurement instruments verified in previous studies related to the TAM, VAM, and CE. Each construct was measured using three items on a five-point Likert scale (1 = strongly disagree, 5 = strongly agree). Table 2 presents an overview of each construct, the number of items, sample items, and their sources. The complete questionnaire (original Chinese version and English back-translation) is included in the Appendix A. To ensure measurement quality, the internal consistency and convergent validity of all reflective constructs were evaluated using Cronbach’s alpha, composite reliability (CR), and average variance extracted (AVE), in line with established guidelines.

**Table 2:** Measurement of constructs.

Construct	Measurement items	Source(s)
PP	[PP_1] The product is priced at a level affordable for ordinary consumers. [PP_2] Considering the safety and quality standards of the product, the price is fair and reasonable. [PP_3] Compared with other substitute products, this product provides satisfactory value for its price.	(28, 29, 60)
AP	[AP_1] The advertisement transparently discloses key information without exaggerating health benefits. [AP_2] The source of the advertisement is trustworthy (e.g., expert/ professional endorsement). [AP_3] The advertisement is clear and easy to understand, enabling informed choices.	(35, 61)
BI	[BI_1] The brand has strong credibility in the health sector. [BI_2] The brand shows responsibility for quality and safety. [BI_3] I trust the brand’s health-related claims.	(39, 62, 63)
IP	[IP_1] The product information is evidence-based and cites authoritative sources. [IP_2] Details about ingredients, eligibility, and risks are complete and clear. [IP_3] I can easily understand the product’s health information.	(19, 23, 42)
PC	[PC_1] The product/service can be personalized to fit my health needs. [PC_2] Personalization respects personal data and explains how the data are used. [PC_3] Personalization improves the fit with my health condition and preferences.	(64, 65)
PQ	[PQ_1] Overall, this product is a reliable, high-quality item. [PQ_2] The product meets expected health and safety standards. [PQ_3] The product’s composition and performance are solid from a professional or user perspective.	(66, 67)
PV	[PV_1] This product is a worthwhile investment for prevention and health management. [PV_2] The overall benefits (health, convenience, reassurance, etc.) exceed the cost I paid. [PV_3] Compared with alternative products, this one provides greater overall value.	(22)
PI	[PI_1] I am willing to use or purchase this product. [PI_2] If possible, I would choose this product. [PI_3] I would recommend this product to others who may benefit from it.	(68)
CE	[CE_1] Purchasing domestic health products helps support the national economy. [CE_2] It is desirable to prioritize domestic health products. [CE_3] I prefer domestic health products over imported ones.	(69–71)
Political identity	“Are you a member of the Chinese Communist Party (CCP)?” (Yes/No)	Self-reported

## 4 Results

### 4.1 Sample characteristics

The final valid sample comprised 875 respondents, and the demographic composition was well balanced across groups. Gender distribution was nearly equal, with 49% male and 51% female respondents, indicating no significant bias. Respondents in their 30s accounted for 34%, followed by those in their 20s (30%), 40s (22%), and 50 years or older (15%), showing adequate representation of young and middle-aged adults—the main consumer groups of healthcare products. Regarding education, 67% held a bachelor’s degree and 20% held a graduate degree or higher, suggesting generally high health literacy and technology acceptance. The majority (40.3%) reported monthly income between 5,000 and 9,999 CNY, indicating predominance of middle-income earners. Additionally, 21% of respondents identified as members of Chinese Communist Party (CCP), which closely aligns with the national average (approximately 21%) and supports the representativeness for political segmentation analyses. Overall, the sample reflects balanced demographic diversity and ensures reliability for subsequent PLS-SEM and MGA. The results are summarized in Table 3.

**Table 3:** Demographic characteristics of respondents (*N* = 875).

Characteristic	Category	Frequency	Percentage (%)
Gender	Male	496	49
	Female	512	51
Age group	18–29 years	301	30
	30–39 years	342	34
	40–49 years	218	22
	50 years and above	147	14
Education level	High school or below	129	13
	College/undergraduate	673	67
	Graduate degree or above	206	20
Monthly income (CNY)	<5,000	245	24
	5,000–9,999	406	40
	10,000–14,999	218	22
	≥15,000	139	14
CCP membership	Member	212	21
	Non-member	796	79

### 4.2 Evaluation of the measurement model

Before testing the structural relationships, the reliability and validity of the measurement model were evaluated. As shown in Table 4, all standardized factor loadings exceeded the recommended threshold value of 0.70 (54), indicating appropriate indicator reliability. The reliability and convergent validity of each latent variable used in this study were verified, and all indicators met the statistical criteria.

**Table 4:** Reliability and validity.

Construct	Item	Factor loading	Cronbach’s *α*	CR	AVE
PP	PP_01	0.948	0.806	0.862	0.679
	PP-02	0.778			
	PP-03	0.731			
AP	AP_01	0.881	0.851	0.91	0.771
	AP_02	0.875			
	AP_03	0.878			
BI	BI_01	0.879	0.856	0.913	0.777
	BI_02	0.877			
	BI_03	0.888			
IP	IP_01	0.895	0.864	0.917	0.786
	IP_02	0.883			
	IP_03	0.882			
PC	PC_01	0.875	0.858	0.913	0.779
	PC_02	0.886			
	PC_03	0.886			
PQ	PQ_01	0.879	0.853	0.911	0.773
	PQ_02	0.873			
	PQ_03	0.885			
PV	PV_01	0.884	0.857	0.913	0.778
	PV_02	0.882			
	PV_03	0.88			
PI	PI_01	0.89	0.865	0.917	0.787
	PI_02	0.881			
	PI_03	0.891			
CE	CE_01	0.899	0.871	0.921	0.795
	CE_02	0.882			
	CE_03	0.894			

All outer loadings exceeded 0.70; AVE > 0.50, and CR > 0.70, confirming convergent validity Hair et al. (55).

First, all factor loadings of each measurement item were above 0.70, confirming that each item stably explained its corresponding construct (55). The Cronbach’s *α* values ranged from 0.806 to 0.871, demonstrating internal consistency for all variables. The CR values ranged from 0.862 to 0.921, showing a high level of reliability (56). The AVE values ranged from 0.679 to 0.795, all exceeding the standard threshold of 0.50, thereby confirming that each latent variable possessed adequate convergent validity (57). These results indicate that all constructs in the research model have statistically reliable measurement stability and conceptual consistency.

Therefore, the measurement model in this study satisfies the basic requirements for conducting the structural model analysis and is judged to have secured sufficient reliability and validity for interpreting the relationships among variables.

Next, Table 5 presents the results of the discriminant validity test conducted according to the Fornell–Larcker criterion (56). The square root of the Average Variance Extracted (√AVE) for each latent variable was greater than the correlation coefficients between that variable and all other variables. For example, the √AVE of AP was 0.878, which was higher than its correlation coefficients with other variables (0.559–0.566). Similarly, the √AVE of PQ was 0.879, which was also greater than the correlations with other constructs. These results indicate that all constructs are mutually distinct and that the measurement model possesses sufficient discriminant validity.

**Table 5:** Discriminant validity (Fornell–Larcker).

Constructs	AP	BI	CE	IP	PC	PI	PP	PQ	PV
AP	0.878								
BI	0.559	0.881							
CE	0.213	0.201	0.892						
IP	0.067	0.069	0.069	0.887					
PC	0.519	0.558	0.182	0.098	0.882				
PI	0.361	0.361	0.292	0.069	0.36	0.887			
PP	0.071	0.063	0.023	0.052	0.063	−0.004	0.824		
PQ	0.566	0.575	0.229	0.099	0.545	0.397	0.049	0.879	
PV	0.411	0.438	0.246	0.1	0.457	0.528	0.009	0.451	0.882

Diagonal values are √AVE. Off-diagonal correlations are below √AVE.

Discriminant validity was further examined using the Heterotrait–Monotrait Ratio (HTMT) criterion to assess the distinctiveness among constructs, and the results are presented in Table 6. The results showed that all HTMT values between constructs were below the conservative threshold of 0.85, confirming that discriminant validity was established (57). In particular, the highest ratios were 0.671 between BI and PQ, 0.664 between AP and PQ, and 0.651 between PC and BI, while most HTMT values generally remained in the range of 0.1–0.6. This indicates that each latent variable is distinguished as an independent construct. Moreover, the HTMT value of the interaction term (CE × PQ) was also very low, below 0.20 in its relationships with all other variables, suggesting that there is no concern regarding multicollinearity or conceptual redundancy.

**Table 6:** Discriminant validity (HTMT).

HTMT ratio	AP	BI	CE	IP	PC	PI	PP	PQ	PV
BI	0.655								
CE	0.248	0.233							
IP	0.078	0.084	0.079						
PC	0.606	0.651	0.21	0.115					
PI	0.421	0.419	0.336	0.08	0.418				
PP	0.077	0.061	0.025	0.067	0.057	0.042			
PQ	0.664	0.671	0.266	0.115	0.636	0.462	0.047		
PV	0.481	0.511	0.285	0.116	0.533	0.613	0.017	0.527	
CE × PQ	0.097	0.094	0.074	0.03	0.04	0.174	0.037	0.105	0.199

HTMT ratios <0.85, confirming discriminant validity.

Next, we conducted a Common Method Bias (CMB) test, and the results are presented in Table 7. According to the full collinearity assessment, all VIF values were below 3.3 (e.g., AP → PQ = 1.602, BI → PQ = 1.699, PQ → PV = 1.063, PV → PI = 1.000, etc.), indicating that there was no potential issue of multicollinearity or CMB (58). In addition, the Harman’s single-factor test showed that the variance explained by a single factor was 34.6%, which is below the 50% threshold, indicating that no single factor was dominant (59). Therefore, it can be concluded that the data used in this study have a very low likelihood of distortion caused by CMB.

**Table 7:** Common method bias test.

Test	Criterion	Result	Interpretation
Full collinearity (VIF)	VIF < 3.3	AP → PQ = 1.602 BI→PQ = 1.699 IP → PQ = 1.012 PC → PQ = 1.603 PP → PQ = 1.008 PQ → PV = 1.063 PV → PI = 1.000 CE × PQ → PV = 1.012	All VIF < 3.3 → No multicollinearity or CMB issue
Harman’s single-factor test	<50% variance explained	34.60%	No single-factor dominance
Kock’s full collinearity test	VIF < 3.3	Satisfied	Acceptable

Next, we analyzed the explanatory power, effect size, and predictive relevance of the structural model (55), and the results are presented in Table 8. The *R*^2^ of PQ was 0.555, indicating that the exogenous variables explained approximately 55.5% of the variance in PQ, while its *Q*^2^ value (0.446) was greater than zero, showing high predictive relevance. Among the predictor variables, AP, BI, and PC showed small effect sizes, whereas IP and PP exhibited statistically negligible effects. The *R*^2^ of PV was 0.343, indicating that PQ, CE, and CE × PQ together explained about 34% of the total variance. The *f*^2^ of PQ (0.204) represented a medium-level effect, while CE and CE × PQ showed small effects. Finally, the *R*^2^ of PI was 0.379, demonstrating a strong explanatory power of PV, and its *f*^2^ (0.387) indicated a large effect size. Since all endogenous variables had *Q*^2^ values greater than zero, the research model was evaluated as having both sufficient predictive relevance and structural robustness overall.

**Table 8:** Model fit, effect sizes, and predictive relevance.

Endogenous construct	*R* ^2^	Predictor	*f*^2^ Effect size	Interpretation	*Q*^2^ (Predictive relevance)
PQ	0.555	AP	0.092	Small	0.446 > 0 → Predictive relevance
		BI	0.085	Small	
		IP	0.003	Negligible	
		PC	0.064	Small	
		PP	0	Negligible	
PV	0.343	PQ	0.204	Medium	0.248 > 0 → Predictive relevance
		CE	0.026	Small	
		CE × PQ	0.024	Small	
PI	0.379	PV	0.387	Large	0.148 > 0 → Predictive relevance

### 4.3 Hypothesis testing

After confirming the reliability and validity of the measurement model, the authors verified the causal relationships of the proposed hypotheses through the structural model analysis. Based on 5,000 bootstrapping resamples, the significance of each path coefficient was evaluated, and most of the major paths in the research model were found to be statistically significant. The results of the hypothesis tests are summarized in Table 9.

**Table 9:** Structural model results.

Hypothesis	Path	*β*	*t*-value	*p*-value	Result
H1	PP → PQ	0.014	0.321	0.748	Not supported
H2	AP → PQ	0.284	7.282	0	Supported
H3	BI → PQ	0.281	6.976	0	Supported
H4	IP → PQ	0.038	1.695	0.09	Not supported
H5	PC → PQ	0.243	2.548	0.011	Supported
H6	PQ → PV	0.435	6.921	0	Supported
H7	PV → PI	0.509	8.493	0	Supported
H8	CE × PQ → PV	0.118	1.832	0.067	*Moderation (p < 0.10)*

Bootstrapping with 5,000 samples; significance levels at 0.01, 0.05, and 0.10. Italicized values indicate statistical significance at the 10% level (*p* < 0.10).

First, regarding the effects of exogenous variables on PQ, Advertising Perception (AP → PQ: *β* = 0.284, *t* = 7.282, *p* < 0.001), Brand Image (BI → PQ: *β* = 0.281, *t* = 6.976, *p* < 0.001), and Personal Customization (PC → PQ: *β* = 0.243, *t* = 2.548, *p* = 0.011) all showed significant positive effects. This suggests that consumers’ perceptions of the quality of health-related products are formed more strongly by emotional and social factors—such as the credibility of advertising messages, the social image of the brand, and personalized service experience—than by functional factors such as price or information. In other words, consumers tend to evaluate the quality of health products through cognitive trust and symbolic value, rather than through objective utility. Therefore, beyond the traditional utility-based explanatory framework of TAM or VAM, this finding shows that in the field of public health communication, a socio-psychological trust base functions as the core of quality evaluation.

In contrast, Information Perception (IP → PQ: *β* = 0.038, *t* = 1.695, *p* = 0.09) and Price Perception (PP → PQ: *β* = 0.014, *t* = 0.321, *p* = 0.748) were not statistically significant, indicating that neither the amount of information nor the level of price has a substantial influence on consumers’ quality perception. This can be interpreted as stemming from the characteristics of health products as credence goods, for which direct verification of quality is difficult. Consumers tend to place more importance on “who speaks (credibility of the source)” and “how well it fits me (personal relevance)” than on the amount of information or price level. These results suggest that traditional marketing strategies centered on information and price may be limited in effectiveness for health and safety product categories, and that communication designs focusing on non-functional elements such as credibility, clarity, and personalization are more effective.

Next, the path PQ → PV (Perceived Quality → Perceived Value) was significant (*β* = 0.435, *t* = 6.921, *p* < 0.001), supporting Hypothesis H6, which posited that consumers’ perceived quality directly affects their overall value evaluation of the product. This result is consistent with the key mechanism proposed in the VAM and demonstrates the process through which product quality is transformed into cognitive value grounded in trust, rather than into mere utility. PV, in turn, had a strong effect on PI. The path coefficient for PV → PI was *β* = 0.509, *t* = 8.493, *p* < 0.001, and H7 was also strongly supported. This shows that a positive perception of product value serves as a major driver leading to consumers’ behavioral intentions, particularly in purchase decisions for health products.

Meanwhile, the moderating effect of CE on the relationship between PQ and PV was tested. The coefficient for the CE × PQ → PV path was *β* = 0.118, *t* = 1.832, *p* = 0.067, showing a weak moderating effect at the 10% significance level (H8). This means that consumers with higher ethnocentrism tend to transfer “the quality of domestic brands” into higher perceived value. Although the magnitude of the moderating effect is not large, this result suggests that cultural-identity factors can exert subtle yet meaningful influences on consumers’ cognitive evaluation structures.

These findings indicate that the integrated TAM–VAM model is valid in the context of healthcare product adoption, and that PQ is formed not merely by technological usefulness but by social trust and personalized experience, thus extending the traditional models one step further. Accordingly, the structural model of this study provides both theoretical integration and practical implications at the intersection of health communication and consumer behavior research, and it can serve as empirical evidence establishing the conceptual foundation for a future “trust-based value formation model.”

### 4.4 Moderating mediation effect of consumer ethnocentrism

After verifying the reliability of the measurement model and the main paths of the structural model, we additionally analyzed the moderated mediation effect of CE. The results estimated through 5,000 bootstrapping resamples are shown in Table 10.

**Table 10:** Moderated mediation model summary.

Indirect Path	*β*	*t*	*p*	Interpretation
PQ → PV → PI	0.221	4.676	0	Significant mediation
CE × PQ → PV → PI	0.041	2.97	0.003	Significant moderated mediation

First, the effect of the basic indirect path (PQ → PV → PI) was significant (*β* = 0.221, *t* = 4.676, *p* < 0.001), confirming that PQ influences PI through the mediation of PV. In other words, the higher consumers evaluate the quality of a health product, the more highly they perceive its overall value, which in turn strengthens their actual purchase intention.

Next, the indirect path including the moderating effect of CE (CE × PQ → PV → PI) was also significant (*β* = 0.041, *t* = 2.970, *p* = 0.003), empirically confirming the presence of a moderated mediation effect, in which CE moderates the mediating pathway among quality, value, and PI. This result means that consumers with higher levels of ethnocentrism tend to transfer their perception of domestic product quality more strongly into PV and PI. That is, as the level of CE increases, the link between quality and value becomes stronger, reflecting a value formation process anchored in cultural identity.

In short, CE functions not merely as a difference in product attitude but as a moderator of the cognitive pathway through which PQ leads to PV and PI. This finding indicates that in the market for credence goods such as healthcare products, national identity and ingroup identification can influence consumers’ value formation and PI, providing meaningful empirical evidence of sociocultural effects in trust-based consumption contexts.

### 4.5 Multi-group analysis

We also conducted a MGA to explore whether political identity (Chinese Communist Party membership, CCP membership) affects the structural relationships within the research model.

Before comparing the two groups (CCP members vs. non-members), we verified the measurement invariance of the indicators by applying the Measurement Invariance of Composite Models (MICOM) procedure proposed by Hair et al. (55) in a stepwise manner. The MICOM procedure consists of three stages: (1) assessment of configural invariance, (2) assessment of mean compositional invariance, and (3) assessment of variance compositional invariance. According to the results, invariance was generally established across all three stages. The detailed results of the MICOM analysis are shown in Table 11.

**Table 11:** Result of MICOM (CCP member/CCP non-member).

Step	Constructs	OR	CPM	*p* value	Constructs	OR	CPM	*p* value
Configural invariance assessment	AP	1	1	0.158	PI	1	1	0.274
	BI	1	1	0.898	PP	0.9	0.721	0.719
	CE	0.999	0.999	0.47	PQ	1	1	0.482
	IP	0.999	0.984	0.914	PV	1	1	0.678
	PC	1	1	0.192				
Mean invariance assessment	AP	−0.033	0.001	0.626	PI	0.18	−0.003	0.008
	BI	−0.014	−0.002	0.856	PP	−0.08	0.001	0.246
	CE	−0.065	−0.003	0.32	PQ	0.002	0	0.987
	IP	0.002	−0.002	0.973	PV	0.046	−0.003	0.514
	PC	0.111	0	0.089				
Variance invariance assessment	AP	0.008	−0.003	0.913	PI	−0.049	−0.004	0.295
	BI	−0.011	0	0.86	PP	−0.027	−0.003	0.78
	CE	0.018	0	0.772	PQ	0.06	−0.001	0.335
	IP	−0.072	−0.002	0.497	PV	0.058	0.001	0.291
	PC	−0.002	0	0.971				

OR, original correlation between composite scores across groups; CPM, correlation permutation mean. A non-significant *p*-value (*p* > 0.05) indicates invariance of the corresponding construct across CCP member and non-member groups.

In the configural invariance stage, the Original Correlation (OR) and Compositional Correlation (CPM) of all latent variables (AP, BI, CE, IP, PC, PQ, PV, PI, etc.) were found to be 1 or close to 1 (all *p*-values > 0.05), indicating that both groups share the same conceptual structure and measurement composition. In the mean invariance stage, most variables showed no significant mean difference (*p* > 0.05). In particular, for the key constructs PQ and PV, the *p*-values were 0.987 and 0.514 respectively, confirming full mean invariance. However, for PI, the *p*-value was 0.008, showing a slightly significant difference. Nevertheless, since the other major constructs showed no statistically significant differences, the overall perceptual structure of the two groups can be regarded as similar. In the variance invariance assessment, the variance differences of all constructs were nonsignificant (*p* > 0.05), showing that the variance structure of the measurement model was also consistent between the two groups.

Taken together, the MICOM results indicate that configural, mean, and variance invariance were all achieved at either partial or full levels between the two groups (CCP members vs. non-members). Therefore, it was confirmed that the comparison of structural models according to political identity is statistically valid.

After establishing measurement invariance through the MICOM analysis, we conducted a MGA to examine whether the structural relationships differ according to political identity (CCP membership). Table 12 presents the main path coefficients (*β*) and their differences (Δ*β*) between the CCP member group and the non-member group.

**Table 12:** Result of MGA (by CCP member).

Path	CCC member *β*	CCC non-member *β*	Δ*β*	*p*-value
PV → PI	0.606	0.446	0.16	0.025
CE × PQ → PV	0.229	0.036	0.193	0.003
PQ → PV → PI	0.28	0.152	0.127	0.008
PC → PQ → PV → PI	0.079	0.029	0.05	0.025

The analysis revealed significant group differences in four key paths. First, for the PV → PI path, the coefficient was *β* = 0.606 for the CCP member group and *β* = 0.446 for the non-member group, and the difference (Δ*β* = 0.160) was statistically significant (*p* = 0.025). This indicates that, compared with non-members, CCP members show a stronger transition from value perception to purchase intention when they perceive higher product value. In other words, consumers with higher political identification exhibit a stronger tendency for value evaluation to translate into behavioral intention. This result can be interpreted as supporting the Social Identity–Behavior Link, which explains how social identity influences behavioral decision-making.

Second, in the CE × PQ → PV path, the CCP member group (*β* = 0.229) showed a much stronger effect than the non-member group (*β* = 0.036), and the difference (Δ*β* = 0.193) was highly significant (*p* = 0.003). This suggests that the stronger the national and political identity among CCP members, the more greatly it reinforces the relationship between quality perception and value evaluation. In other words, even at the same quality level, consumers with high political identification tend to assign greater value to “domestic products” or “public health campaign products.” This result shows that in the mediating–moderating process of CE on the PQ–PV relationship, political identity salience can act as an additional amplifying factor.

Third, the indirect path PQ → PV → PI was also significantly higher for CCP members (*β* = 0.280) than for non-members (*β* = 0.152), with a difference of Δ*β* = 0.127 (*p* = 0.008). This means that the mediating effect linking perceived quality to value and purchase intention operates more strongly among CCP members. In other words, CCP members have a more solidly formed psychological transfer mechanism that converts quality into value and value into behavior. This phenomenon can be interpreted as resulting from consumers with higher political affiliation placing greater trust in group policies or public-interest messages, such that political trust acts as an additional mediating reinforcement factor in the process by which quality perception transfers into value.

Fourth, a significant difference was also observed in the complex indirect path PC → PQ → PV → PI (Δ*β* = 0.050, *p* = 0.025). This shows that the overall pathway from PC through PV, and PI operates more strongly in the CCP member group. In other words, CCP members interpret personalized product experiences or customized communication as an extension of collective identity and public messaging, thereby activating a politico-psychological dual mechanism that simultaneously satisfies personal satisfaction and group belongingness.

These results indicate that political and ideological factors can strengthen the process of cognitive–affective–social value transfer in individuals’ consumer decision-making.

## 5 Discussion and conclusion

### 5.1 Summary of research

This study viewed healthcare products as credence goods and empirically tested a trust-based value acceptance model that integrates the TAM and the VAM. The analysis results strongly supported the sequential path PQ → PV → PI, and the mediating effect of PV showed greater explanatory power than the direct effect of PQ. AP, BI, and PC significantly increased PQ, whereas PP and IP had minimal effects. CE functioned as a cultural trust heuristic, strengthening the PQ–PV relationship, and the MGA results showed that Political Identity (CCP membership) significantly heightened effects in the paths PV → PI, CE × PQ → PV, PQ → PV → PI, and PC → PQ → PV → PI for the member group. This suggests that political identification acts as an institutional trust factor that amplifies the strength of value transfer. In sum, the acceptance of healthcare products is not determined by mere technological utility but by value judgments grounded in trust, safety, and identity.

### 5.2 Theoretical implications

This study proposed a new theoretical framework to explain consumers’ behavioral intentions in the domain of credence goods such as healthcare products, by integrating the TAM and the VAM.

First, while the traditional TAM has focused on functional utility, such as PU and PEU, this study theoretically supports the transition from technology acceptance to value acceptance by expanding TAM toward value-based evaluation. Because the objective verification of quality prior to purchase is difficult in healthcare products, consumers evaluate mainly in terms of trustworthiness, safety, and authenticity of information. Under such conditions, the integration of TAM and VAM empirically demonstrates that the value formation process involves not only cognitive utility but also factors of trust, safety, and identity. This finding is consistent with value-based adoption research, which identifies perceived value as the most proximal determinant of behavioral intention, particularly in high-uncertainty and credence-good contexts (28, 29).

Second, this study verified the applicability of TAM in the context of credence goods such as healthcare and health-related technologies. While traditional theories such as the TPB or the TRA explain behavioral intention primarily through attitudes and norms, TAM predicts behavior through cognitive processes of technological attributes and quality perceptions. The findings of this study show that such a structure remains valid for technology-intensive products such as health management technologies, functional foods, and digital health devices. That is, consumers form purchase intentions not merely from attitudes or norms but from trust-based evaluations of quality and the perceived value derived from them. This supports prior arguments that TAM can be theoretically extended when quality uncertainty and risk perceptions are central to decision-making (9, 20). In this sense, TAM is demonstrated to be a theoretically extendable framework applicable to the field of public health.

Third, this study redefined the theoretical role of CE. Unlike prior studies that treated CE as a direct predictor of attitudes or PI, this research positioned CE as a moderating factor that strengthens the relationship between PQ and PV. This explains how consumers convert quality evaluations into value judgments through ingroup identity and cultural trust. Accordingly, the study extends CE research beyond country image or country-of-origin effects, framing it instead as a cultural trust mechanism for value formation. This reconceptualization aligns with ethnocentrism research that frames CE as a moral and cultural trust heuristic embedded in social identity (48, 49).

Fourth, the exploratory introduction of Political Identity (CCP membership) proposes a social identity–based extension of public health communication theory. The results of the MGA showed that the member group had a stronger chain effect in the PQ → PV → PI path than the non-member group, and an enhanced moderating effect in the CE × PQ → PV path. This demonstrates that political identity is not merely an ideological attitude but a social trust factor reflecting institutional alignment and trust in the national system. In other words, institutional trust functions as a social identity–based trust mechanism that strengthens the link between value formation and behavioral intention. This interpretation is consistent with prior studies suggesting that institutional and political alignment enhances trust in official communication and policy-driven health initiatives (44, 45).

In conclusion, by integrating TAM and VAM into the field of public health consumption, this study theoretically establishes the connected pathways among technology acceptance, value acceptance, and trust formation. This presents a new theoretical perspective that, in domains characterized by high uncertainty and perceived risk—such as healthcare products—consumer decision-making is shaped not by simple utility but by trust- and identity-based value evaluations.

### 5.3 Practical and public health implications

The results of this study provide practical implications for both consumer communication strategies of healthcare products and public health policies.

First, the results confirmed that PQ and PV form the core mediating pathway leading to PI. This implies that strategies focusing on ensuring information reliability, clarity, and sufficiency, and strengthening brand credibility and transparency, are more effective than those centered on price competition or sales promotion. Since consumers in the healthcare product market make purchasing decisions based more on trust than on cost, firms should redesign their advertising, branding, and information dissemination into trust-building messages.

Second, this study showed that personalization strengthens the transfer path among quality, value, and purchase intention. This finding provides a key implication for the digital health era: both businesses and policymakers should consider privacy protection and trust assurance simultaneously when designing data-driven personalized services. Providing personalized health information and tailored nutrition or exercise recommendations delivers not only convenience but also psychological trust, and can serve as a facilitating factor that enhances the continuity of health behavior even in the public sector. This aligns with previous studies indicating that personalization enhances perceived relevance and trust only when supported by transparent data governance (44).

Third, the finding that CE significantly strengthened the relationship between PQ and PV suggests that public communication can be more effective when it utilizes domestic quality standards, national certification, and public trust frames. In particular, in national health promotion campaigns such as Healthy China 2030, narrative messages such as “nationally certified” or “protecting citizens’ health” may positively influence value evaluations. This indicates that public-interest brands and health campaigns should strategically employ a cultural trust frame in their communication. Such culturally embedded trust framing has been shown to enhance acceptance of health-related products and messages in prior research (49).

Fourth, the result showing that Political Identity (CCP membership) strengthened the PQ → PV and PV → PI paths demonstrates that institutional trust exerts a substantial influence on the acceptance of public health communication. Accordingly, when governments or public institutions deliver health-related messages, communication should emphasize policy trust, risk management capability, and moral responsibility—beyond the mere provision of information. This approach does not serve as political propaganda but can be understood as a process of building a public trust governance model grounded in citizen confidence. This supports the view that institutional trust functions as a governance mechanism facilitating public compliance and preventive health behavior (45).

Finally, our findings suggest the necessity to redefine healthcare product communication not merely as marketing activity but as a form of public health intervention. Reliable health information, strengthened brand credibility, and the expansion of personalized preventive services can contribute not only to changes in individual health behaviors but also to the reduction of social costs. Therefore, future policy should aim to promote a transition from market-driven consumption stimulation to preventive health consumption.

### 5.4 Limitations and future research directions

Although this study presented an integrated pathway of trust and value formation in healthcare product consumption, several theoretical extensions remain possible. First, the proposed model focused mainly on cognitive factors; therefore, future studies should expand the explanatory power by incorporating emotional and normative variables, such as affective trust and risk perception. Second, the moderating effects of political identity and CE may depend on a given country’s level of institutional trust, making cross-cultural validation meaningful to identify the boundary conditions of the trust mechanism. Third, future research could go beyond survey-based perceptual data by combining actual behavioral data and digital interaction analysis to elucidate the dynamic process of trust and value transfer more comprehensively.

## 6 Conclusion

This study integrated the TAM and the VAM to empirically identify how message cues shape Chinese consumers’ responses to healthcare products. The analysis revealed that trustworthy information, brand credibility, advertising reliability, and personal customization significantly enhanced PQ, and this perception of quality formed the key pathway toward PI through PV. Among all factors, PV was identified as the most influential determinant in health-related decision-making. Moreover, CE strengthened the linkage between quality and value, and political identity partially moderated this relationship, demonstrating the role of institutional trust. These results highlight the need to reinterpret healthcare consumption not as an issue of technological utility alone but as a complex mechanism of trust, value, and identity.

Furthermore, this study holds academic significance by extending technology acceptance theory into the domain of credence goods and theoretically integrating the moderating effects of sociocultural factors within public health communication. From a policy perspective, the findings suggest that communication strategies characterized by evidence-based messaging, transparency, and cultural attunement are crucial pathways for achieving the preventive health goals of Healthy China 2030. Such an approach contributes to strengthening public health governance built upon citizen trust and to fostering a closer connection between individual actions and societal health objectives.

## Data Availability

The raw data supporting the conclusions of this article will be made available by the authors, without undue reservation.

## Footnotes

1
https://www.wjx.cn/
