# Supplementary material for: Consumer ethnocentrism and political identity in value formation within public health communication for healthcare products: evidence from China
Source: Front Public Health. 2026 Jan 14;13:1693501. doi: 10.3389/fpubh.2025.1693501 (PMC12847334; doi:10.3389/fpubh.2025.1693501)
Supplement: Supplementary file 1 [file Table_1.docx]

Appendix A.

**《健康产品消费者感知与健康传播调查问卷》**

| **研究说明与知情同意**  本研究旨在了解健康传播线索（如广告透明度与可信度、品牌公信力、信息清晰度与证据支持、个性化服务与健康适配性、价格的可负担性与公平性）如何影响公众对健康相关产品（预防性保健食品、营养补充剂、数字健康设备等）的感知质量与感知价值，并进一步影响使用/购买意愿。同时，我们考察消费者民族中心主义与政治身份在上述关系中的调节作用，以为预防为主的公共卫生传播策略提供依据。  本研究遵循《赫尔辛基宣言》。问卷匿名，仅用于学术研究，不收集可识别个人信息。参与完全自愿，可随时退出。继续作答即视为您已阅读并同意上述说明。 |
| --- |
| **作答说明**  - 量表：1–5分（1=非常不同意；2=不同意；3=一般；4=同意；5=非常同意）  - 请每题仅勾选一项 |

**筛选题**

在过去12个月中，您是否购买或认真考虑过健康相关产品（如保健食品/营养补充剂/数字健康设备等）？

| 选项 | 勾选 |
| --- | --- |
| 是 | ☐ |
| 否（选择“否”将结束问卷） | ☐ |

**正式题项（1=非常不同意 … 5=非常同意）**

**请在每行对应的同意程度栏内勾选一项。**

| 构念 | 题项编号 | 题目表述 | 1 | 2 | 3 | 4 | 5 |
| --- | --- | --- | --- | --- | --- | --- | --- |
| 价格感知（PP） | PP1 | 该产品的价格对普通消费者是可负担的。 | ☐ | ☐ | ☐ | ☐ | ☐ |
|  | PP2 | 相比其安全与质量标准，该产品定价公平合理。 | ☐ | ☐ | ☐ | ☐ | ☐ |
|  | PP3 | 与同类健康产品相比，该产品性价比令人满意。 | ☐ | ☐ | ☐ | ☐ | ☐ |
| 广告感知（AP） | AP1 | 广告中关键信息透明，未夸大健康功效。 | ☐ | ☐ | ☐ | ☐ | ☐ |
|  | AP2 | 广告来源可信（如专家/权威机构背书）。 | ☐ | ☐ | ☐ | ☐ | ☐ |
|  | AP3 | 广告内容清晰易懂，便于做出知情选择。 | ☐ | ☐ | ☐ | ☐ | ☐ |
| 品牌形象（BI） | BI1 | 该品牌在健康领域的公信力较强。 | ☐ | ☐ | ☐ | ☐ | ☐ |
|  | BI2 | 该品牌对质量与安全负有明确责任。 | ☐ | ☐ | ☐ | ☐ | ☐ |
|  | BI3 | 我信任该品牌的健康相关声明。 | ☐ | ☐ | ☐ | ☐ | ☐ |
| 信息感知（IP） | IP1 | 产品信息基于证据，并引用权威来源。 | ☐ | ☐ | ☐ | ☐ | ☐ |
|  | IP2 | 成分/适用人群/风险提示等信息完整且清晰。 | ☐ | ☐ | ☐ | ☐ | ☐ |
|  | IP3 | 我能轻松理解该产品的健康相关信息。 | ☐ | ☐ | ☐ | ☐ | ☐ |
| 个性化定制（PC） | PC1 | 该产品/服务能依据个人健康需求进行个性化。 | ☐ | ☐ | ☐ | ☐ | ☐ |
|  | PC2 | 个性化过程尊重隐私并说明数据使用。 | ☐ | ☐ | ☐ | ☐ | ☐ |
|  | PC3 | 个性化功能提升了我与自身健康状况的匹配度。 | ☐ | ☐ | ☐ | ☐ | ☐ |
| 感知质量（PQ） | PQ1 | 我认为该产品整体质量可靠。 | ☐ | ☐ | ☐ | ☐ | ☐ |
|  | PQ2 | 该产品符合健康与安全规范的期望。 | ☐ | ☐ | ☐ | ☐ | ☐ |
|  | PQ3 | 从专业与使用角度看，该产品做工/性能过硬。 | ☐ | ☐ | ☐ | ☐ | ☐ |
| 感知价值（PV） | PV1 | 该产品对预防与健康管理是值得的投入。 | ☐ | ☐ | ☐ | ☐ | ☐ |
|  | PV2 | 综合收益（健康、便利、安心）大于我付出的成本。 | ☐ | ☐ | ☐ | ☐ | ☐ |
|  | PV3 | 相较替代方案，该产品更有整体价值。 | ☐ | ☐ | ☐ | ☐ | ☐ |
| 购买/使用意愿（PI） | PI1 | 未来我愿意使用/购买该产品。 | ☐ | ☐ | ☐ | ☐ | ☐ |
|  | PI2 | 若条件允许，我会优先选择该产品。 | ☐ | ☐ | ☐ | ☐ | ☐ |
|  | PI3 | 我愿意将该产品推荐给需要的人。 | ☐ | ☐ | ☐ | ☐ | ☐ |
| 消费者民族中心主义（CE） | CE1 | 购买国产健康产品有助于支持本国经济。 | ☐ | ☐ | ☐ | ☐ | ☐ |
|  | CE2 | 在健康产品上优先支持国产是合乎情理的。 | ☐ | ☐ | ☐ | ☐ | ☐ |
|  | CE3 | 与进口相比，我更愿意选择国产健康产品。 | ☐ | ☐ | ☐ | ☐ | ☐ |

**政治身份（单选）**

| 题目 | 选项 | 勾选 |
| --- | --- | --- |
| 您是中国共产党党员吗？ | 是 | ☐ |
|  | 否 | ☐ |

**人口统计学信息（单选/单填）**

| 题目 | 选项 | 勾选 |
| --- | --- | --- |
| 性别 | 男 ☐　女 ☐ |  |
| 年龄 | 18–29 ☐　30–39 ☐　40–49 ☐　50+ ☐ |  |
| 受教育程度 | 高中及以下 ☐　本/专科 ☐　研究生及以上 ☐ |  |
| 月收入（人民币） | <5,000 ☐　5,000–9,999 ☐　10,000–14,999 ☐　≥15,000 ☐ |  |

Appendix B.

**< Survey on Consumer Perceptions and Health Communication of Healthcare Products >**

| Study Background & Consent  This survey examines how health communication cues—advertising transparency and credibility, brand trustworthiness, clarity and evidence-basis of product information, personalization and health fit, and price affordability/fairness—shape the perceived quality and perceived value of health-related products (e.g., preventive/functional foods, supplements, digital health devices), and, in turn, intention to use/purchase. We also assess the moderating roles of consumer ethnocentrism and political identity to inform prevention-oriented public health communication.  The survey is anonymous, voluntary, and conducted under the Declaration of Helsinki. No personally identifiable data are collected. Continuing indicates informed consent. |
| --- |
| Instructions  Scale: 1–5 (1=Strongly disagree; 2=Disagree; 3=Neutral; 4=Agree; 5=Strongly agree)  Select one option per item. |

**Screening**

In the past 12 months, have you purchased or seriously considered health-related products?

| Option | Check |
| --- | --- |
| Yes | ☐ |
| No (terminates survey) | ☐ |

**Main Items (1=Strongly disagree … 5=Strongly agree)**

**Tick one box per row.**

| Construct | Code | Item wording | 1 | 2 | 3 | 4 | 5 |
| --- | --- | --- | --- | --- | --- | --- | --- |
| Price Perception (PP) | PP1 | The product is affordable for typical consumers. | ☐ | ☐ | ☐ | ☐ | ☐ |
|  | PP2 | Relative to its safety and quality standards, the price is fair and reasonable. | ☐ | ☐ | ☐ | ☐ | ☐ |
|  | PP3 | Compared with alternatives, the product offers satisfactory value-for-money. | ☐ | ☐ | ☐ | ☐ | ☐ |
| Advertising Perception (AP) | AP1 | Advertising discloses key information transparently without overstating health benefits. | ☐ | ☐ | ☐ | ☐ | ☐ |
|  | AP2 | The advertising source is credible (e.g., expert/authoritative endorsement). | ☐ | ☐ | ☐ | ☐ | ☐ |
|  | AP3 | The advertising is clear and easy to understand for informed choice. | ☐ | ☐ | ☐ | ☐ | ☐ |
| Brand Image (BI) | BI1 | The brand has strong public credibility in health. | ☐ | ☐ | ☐ | ☐ | ☐ |
|  | BI2 | The brand shows accountability for quality and safety. | ☐ | ☐ | ☐ | ☐ | ☐ |
|  | BI3 | I trust the brand’s health-related claims. | ☐ | ☐ | ☐ | ☐ | ☐ |
| Information Perception (IP) | IP1 | Product information is evidence-based and cites authoritative sources. | ☐ | ☐ | ☐ | ☐ | ☐ |
|  | IP2 | Details on ingredients/eligibility/risks are complete and clear. | ☐ | ☐ | ☐ | ☐ | ☐ |
|  | IP3 | I can easily understand the product’s health information. | ☐ | ☐ | ☐ | ☐ | ☐ |
| Personal Customization (PC) | PC1 | The product/service allows personalization to my health needs. | ☐ | ☐ | ☐ | ☐ | ☐ |
|  | PC2 | Personalization respects privacy and explains data use. | ☐ | ☐ | ☐ | ☐ | ☐ |
|  | PC3 | Personalization improves fit with my health status/preferences. | ☐ | ☐ | ☐ | ☐ | ☐ |
| Perceived Quality (PQ) | PQ1 | Overall, the product is reliably high-quality. | ☐ | ☐ | ☐ | ☐ | ☐ |
|  | PQ2 | The product meets expected health and safety standards. | ☐ | ☐ | ☐ | ☐ | ☐ |
|  | PQ3 | From professional/use perspectives, build/performance is sound. | ☐ | ☐ | ☐ | ☐ | ☐ |
| Perceived Value (PV) | PV1 | For prevention and health management, this is a worthwhile investment. | ☐ | ☐ | ☐ | ☐ | ☐ |
|  | PV2 | Overall benefits (health, convenience, reassurance) exceed my costs. | ☐ | ☐ | ☐ | ☐ | ☐ |
|  | PV3 | Versus alternatives, this product delivers greater overall value. | ☐ | ☐ | ☐ | ☐ | ☐ |
| Purchase/Use Intention (PI) | PI1 | I intend to use/purchase this product. | ☐ | ☐ | ☐ | ☐ | ☐ |
|  | PI2 | If feasible, I would choose this product. | ☐ | ☐ | ☐ | ☐ | ☐ |
|  | PI3 | I would recommend it to others who may benefit. | ☐ | ☐ | ☐ | ☐ | ☐ |
| Consumer Ethnocentrism (CE) | CE1 | Buying domestic health products helps support the national economy. | ☐ | ☐ | ☐ | ☐ | ☐ |
|  | CE2 | Prioritizing domestic health products is appropriate. | ☐ | ☐ | ☐ | ☐ | ☐ |
|  | CE3 | I prefer domestic over imported health products. | ☐ | ☐ | ☐ | ☐ | ☐ |

**Political Identity (single choice)**

| Item | Option | Check |
| --- | --- | --- |
| Are you a CCP member? | Yes | ☐ |
|  | No | ☐ |

**Demographics**

| Item | Options | Check |
| --- | --- | --- |
| Gender | Male ☐　Female ☐ |  |
| Age | 18–29 ☐　30–39 ☐　40–49 ☐　50+ ☐ |  |
| Education | High school or below ☐　Undergraduate ☐　Graduate or above ☐ |  |
| Monthly income (CNY) | <5,000 ☐　5,000–9,999 ☐　10,000–14,999 ☐　≥15,000 ☐ |  |

Appendix C.

Supplementary Table S1 reports the Fornell–Larcker criterion. In all cases, the square roots of the AVE (diagonal values) exceeded the inter-construct correlations, confirming discriminant validity.

Table S1. Fornell-Larcker Result

|  | AP | BI | CE | IP | PC | PI | PP | PQ | PV |
| --- | --- | --- | --- | --- | --- | --- | --- | --- | --- |
| AP | 0.839 |  |  |  |  |  |  |  |  |
| BI | 0.382 | 0.846 |  |  |  |  |  |  |  |
| CE | 0.341 | 0.382 | 0.853 |  |  |  |  |  |  |
| IP | 0.331 | 0.356 | 0.358 | 0.845 |  |  |  |  |  |
| PC | 0.288 | 0.354 | 0.303 | 0.34 | 0.839 |  |  |  |  |
| PI | 0.181 | 0.204 | 0.196 | 0.167 | 0.157 | 0.825 |  |  |  |
| PP | -0.036 | -0.01 | 0.008 | -0.04 | 0.027 | 0.014 | 0.815 |  |  |
| PQ | 0.352 | 0.367 | 0.372 | 0.323 | 0.302 | 0.228 | -0.04 | 0.838 |  |
| PV | 0.242 | 0.265 | 0.209 | 0.269 | 0.277 | 0.182 | 0.009 | 0.24 | 0.843 |

Note. Diagonal values (√AVE) greater than inter-construct correlations indicate discriminant validity

Supplementary Table S2 presents the HTMT ratios, all of which were below the conservative threshold of 0.85, further supporting discriminant validity.

Table S2. HTMT Result

|  | AP | BI | CE | IP | PC | PI | PP | PQ | PV | CE x PQ |
| --- | --- | --- | --- | --- | --- | --- | --- | --- | --- | --- |
| AP |  |  |  |  |  |  |  |  |  |  |
| BI | 0.478 |  |  |  |  |  |  |  |  |  |
| CE | 0.425 | 0.472 |  |  |  |  |  |  |  |  |
| IP | 0.417 | 0.441 | 0.445 |  |  |  |  |  |  |  |
| PC | 0.360 | 0.442 | 0.373 | 0.426 |  |  |  |  |  |  |
| PI | 0.230 | 0.258 | 0.248 | 0.212 | 0.203 |  |  |  |  |  |
| PP | 0.044 | 0.049 | 0.023 | 0.065 | 0.083 | 0.048 |  |  |  |  |
| PQ | 0.443 | 0.458 | 0.464 | 0.405 | 0.379 | 0.293 | 0.047 |  |  |  |
| PV | 0.302 | 0.330 | 0.259 | 0.336 | 0.346 | 0.232 | 0.049 | 0.304 |  |  |
| CE x PQ | 0.173 | 0.080 | 0.073 | 0.090 | 0.015 | 0.075 | 0.037 | 0.043 | 0.088 |  |

Note: HTMT values < 0.85, supporting discriminant validity.
